# Cremation or "Decent Burial"?

**Published:** 1890-05-24

**Authors:** 


### The Doctor's Pulpit.

### CREMATION OR "DECENT BURIAL?"

"An old man, broken with the storms of state,"
"Is come to lay his weary bones among ye;"
"Give him a little earth for charity."

There is satisfaction in the thought that Wolsey was not cremated. If any of the little dogs of science are inclined to set up disapproving howls and barks at such flagrant heresy, we cry them peace. The world was not made yesterday; England has known great ages other than the Victorian; men have lived and thought before the birth of nineteenth century chemists; Bacon had as much science in his one small head as all the mechanical microscopists and routine experimenters of the present time put together. Science is not a mere question of the microscope, the dissecting knife, and the crucible. It demands vision, range, generalising power, knowledge of what has been, insight into what may be, intellectual greatness and majesty.

This preface is a defensive outwork thrown up to guard what many medical men will consider a retrograde and unscientific position. That position consists in an invincible preference for decent burial as against cremation. To science in itself we are profoundly and absolutely loyal. But to infallible popes in science we have as rooted and as incurable an objection as to infallible popes in politics or religion. We would go so far as to say that it is better to have less science and to hold it with a free and royal mind, than to have more and to be crushed into intellectual enslavement by it. We are by no means convinced, however, that even from a high scientific point of view cremation is better than burial. It may be, nay it is, to be very much preferred to burial as ordinarily carried out in this country. But science herself comes to the help of those who wish to lay their dead decently in the ground instead of consigning them to immediate destruction by fire. She shows us how we may bury our dead even more decently and more reverently than at present, and yet do no more harm to the living than if we cremated them.

In a small country with a large population it is absolutely essential to make the safety of the living a prime consideration in dealing with the question of the methods of interment of the dead. Sir Lyon Playfair is as sober and reliable a scientific authority as any man of science of the present day. He is in fact more reliable than most men who can reasonably claim to be scientists. For whilst he is a man of the highest scientific knowledge he is also a man of broad general culture and of great experience in public affairs. Many men of excellent scientific education lay hold of a fact or principle exactly as a mastiff seizes upon a midnight marauder. To the mastiff, at the moment, there is nothing at all else in the world but himself and the captured thief. He holds on like death itself. Exactly so behaves the inexperienced scientist. He has laid hold of a fact or a principle; it may be after much labour. He knows it is a fact exactly as the mastiff knows that the burglar's calf is real and solid flesh. To him the world consists of himself, his fact or principle, and a certain number of human creatures who are to be pursuaded or compelled into acceptance of his discovery on pain of his most withering and eternal contempt.

Such a man of science, with so little knowledge of the infinite variety of facts and principles that lie outside his own little experience, cannot be accepted as a guide in the practical affairs of life. Such a man of science Sir Lyon Playfair is not! What does Sir Lyon Playfair say on the important point of burial as against cremation? "Burial," he says, "when properly performed, is as innocuous a mode of disposing of the dead as burning." And what does he consider the essential conditions of proper burial? They are three: first, the coffin must be of a perishable nature: second, the soil in which the grave is dug must be dry and porous: third, the graves must not be too crowded. Supposing all these three conditions to be properly fulfilled, what happens to the bodies of the dead? In the short space of three or four years they are resolved into two substances, air and ashes. What is equally important, they are as completely disposed of in this way as if they were burned in a furnace, and that without any possible chance of injury to the living. To quote Sir Lyon Playfair's own words.—"It is now known with certainty that dead matter of all organized beings passes into air for the most part, and a small remnant into ashes which remain in the soil. The changed products of the dead—carbonic acid water, and ammonia—are the food of plants, which, under the action of the sun, mould them into new forms of organic life. Death thus becomes the source of life, and the dissolution of one generation is actually necessary for the support of a succeeding one. The air, the all-abounding air, into which pass all the waste of the living and all the products of the dead, thus becomes the grave of organic death and the cradle of organic life. . . The materials of our living bodies have mainly come from the air, and to the air they must return." Bodies decomposing in dry porous soils at a sufficient depth do not give off to the atmosphere large quantities of offensive and injurious gases, but harmless exhalations of the three common bodies, carbonic acid, water, and ammonia. Even these, as Sir Lyon Playfair points out, are promptly laid hold of by plants for their nourishment and the building up of their tissues.

A very large proportion of English speaking people, and particularly those who have an assured conviction of a final resurrection, entertain the profoundest feelings of aversion to cremation as a method of disposing of the dead. At the same time an intelligent sense of duty to the living causes them to be harrassed with semi-scientific doubts. We have endeavoured to show them that there is a way of burial which is as safe and wholesome for the living as cremation. If readers are convinced by what has been said, a twofold necessity will henceforth be laid upon them: If they have dead of their own to bury they will as far as possible bury them according to the conditions here laid down—that is to say, in perishable coffins, in dry, porous soil, and in a not too crowded graveyard. They will also consider it an imperative duty to explain to their friends the essentials of healthy burial.

In London it must be admitted, and in other large towns, there is some difficulty in carrying out funerals under the conditions described as essential to the health of the living. But the Funeral Reform Association has so far succeeded in gaining the public ear that business persons have found it profitable to provide for the necessities of those who wish to bury their dead without infringing sanitary laws. The London Necropolis Company, whose offices are at 2 Lancaster Place, Strand, have, in their Cemetery at Woking, a perfectly dry porous soil and ample space. They also use coffins made of pulp which break down rapidly in the ground and permit of the natural decomposition of the dead body in the course of three or four years. The Company, we believe, take charge of funerals in exactly the same way as an ordinary undertaker. The pulp of which the coffin is made is very cheap, and this enables funerals to be carried out at much less than the usual cost. It cannot be denied that in populous cities the question of the disposal of the dead is one of the first importance. Indifference on the part of the public is both stupid and criminal. So long as cremation was believed to be the only alternative to ordinary burial people might be excused if they declined to depart from old customs. But now that it is becoming generally recognized that a particular mode of burial satisfies all the conditions of sanitary science as completely as they are satisfied by cremation it cannot be doubted that that method of burial should alone be used by all those to whom cremation is distasteful and practically impossible.

